# Driver license renewal policies and fatal crash involvement rates of older drivers, United States, 1986–2011

**DOI:** 10.1186/s40621-014-0025-0

**Published:** 2014-10-24

**Authors:** Brian C Tefft

**Affiliations:** AAA Foundation for Traffic Safety, 607 14th Street NW, Suite 201, Washington, 20005 DC USA

**Keywords:** Motor vehicle, Traffic, Elderly

## Abstract

**Background:**

Older drivers experience elevated risk of motor vehicle crash involvement, injury, and death. Several states attempt to address these risks through driver license renewal policies; however, little is known about their effects.

**Methods:**

Data from 46 U.S. states from years 1986–2011 were examined. Associations between driver licensing policies and population-based fatal crash involvement rates of drivers aged 55 years and older, in 5-year age groups, were estimated using population-averaged negative binomial regression. Estimates were adjusted for seasonality, time trends, other traffic safety laws, and economic factors. Ratios of relative risks (RRR), which compared changes in fatal crash involvement rates of older drivers associated with changes in licensing policies to corresponding changes in fatal crashes of drivers ages 40–54, were computed to account for other possible sources of confounding.

**Results:**

Mandatory in-person renewal was associated with a 31% reduction in the fatal crash involvement rates of drivers ages 85 and older (RRR: 0.69, 95% Confidence Interval [CI]: 0.48–0.97). When in-person renewal was not required, requiring drivers to pass a vision test was associated with a similar reduction for drivers ages 85+ (RRR: 0.64, 95% CI: 0.49–0.85). When in-person renewal was required, however, requiring a vision test was not associated with any additional reduction, nor was requiring a knowledge test or an on-road driving test. Requiring more frequent license renewal and requiring healthcare providers to report concerns about patients’ driving ability to licensing authorities were not associated with statistically significant reductions in fatal crash involvement rates of older drivers. No policy examined was found to have a significant impact on fatal crash involvement of drivers younger than 85.

**Conclusions:**

Requiring drivers to renew their license in person, or to pass a vision test if not renewing in person, was associated with significant reductions in population-based fatal crash involvement rates for drivers ages 85 and older. The study could not determine how these effects were achieved, for example by specifically removing unsafe older drivers from the driving population or by fostering premature driving cessation. Other policies examined were not found to reduce fatal crash involvement rates of older drivers.

## 1 Background

Although older drivers pose less risk to other people outside of their vehicles than young drivers do; their risk of crash involvement increases somewhat beyond approximately age 70–75 years, and risk of injury or death in the event of a crash increases substantially beyond this age range (Tefft [2008]).

In 2012, 29 million US residents, representing approximately 9% of the population, were 70 years old or older (Ortman et al. [2014]). As older Americans’ share of the population continues to grow, several jurisdictions in the US and abroad are attempting to address the risks associated with aging drivers through a variety of laws and policies related to driver licensing and license renewal. For example, 19 US states currently require drivers over a specific age to renew their licenses more frequently than younger drivers do, 15 states do not allow drivers over a certain age to renew their license by mail and/or online despite allowing younger drivers to do so, and one state requires drivers over the age of 75 years to pass an on-road driving test when renewing their license (Insurance Institute for Highway Safety [2014]).

Despite substantial interest in addressing the safety of older drivers through the driver licensing system, few studies have examined the safety impacts of driver licensing laws and policies for older drivers (Dugan et al. [2013]). Two studies analyzed data from the United States from the 1980’s and concluded that requiring older drivers to pass a vision test to renew their license was associated with reductions in rates of fatal crashes (Levy et al. [1995]; Shipp [1998]). However, a more recent study found that requiring drivers aged 85 years old and older to renew their license in person was associated with reductions in rates of fatal crashes, but requiring a vision test was not associated with further reductions after controlling for mandatory in-person renewal (Grabowski et al. [2004]). The objective of this study was to examine the relationship between state driver license renewal laws and the fatal crash involvement rates of older drivers while controlling for the effects of other factors that might also influence safety.

## 2 Methods

### 2.1 Study design

A panel data set of drivers involved in fatal motor vehicle crashes, driver license renewal policies, and potential confounding variables by state, year, quarter, and age group, spanning years 1986–2011, was compiled and analyzed. Drivers were categorized into age groups 55–59, 60–64, 65–69, 70–74, 75–79, 80–84, and 85+ years. Drivers aged 40–54 years were included as a reference group.

### 2.2 Data

#### 2.2.1 Fatal crashes

Data on drivers involved in fatal motor vehicle crashes were obtained from the National Highway Traffic Safety Administration’s Fatality Analysis Reporting System ([2013]), which comprises data on all motor vehicle crashes that occur on public roadways in the United States and result in a death within 30 days of the crash. Data from crashes that occurred in years 1986–2011 were analyzed. Only crash-involved drivers operating a car, pickup truck, van, minivan, or sport utility vehicle were examined; drivers of motorcycles, large trucks, and other types of vehicles were excluded, as many of these vehicles require a different type of license or in some cases no license and were outside of the scope of the study. Drivers were grouped by the state in which their license was issued (or state of residence if unlicensed), which was not necessarily the state in which the crash occurred.

#### 2.2.2 Driver licensing laws and policies

Historical data on driver license renewal requirements were compiled by review of existing archival sources (Carr et al. [2010]; Federal Highway Administration, [1984], [1986], [1988], [1990], [1992], [1994], [1996]; Hawley and Tannahill [1989]; Lococo [2003]; National Conference of State Legislatures [2005], [2006], [2007], [2008], [2009], [2010], [2011], [2012]; National Highway Traffic Safety Administration & American Association of Motor Vehicle Administrators [1986], [1990], [1995], [1999]; Petrucelli and Malinowski [1992]; Wang et al. [2003]).

Data compiled from these sources included the length of the renewal period; whether renewal must be performed in person or whether renewal by mail or online was allowed; whether visual acuity tests (hereafter *vision tests*), knowledge tests (beyond identification of road signs), and on-road driving tests were required; and whether physicians were required to report patients to the licensing authority in the event of specific medical diagnoses and/or general concerns about driving (hereafter *mandatory reporting*) or whether such reporting was voluntary.

These variables were coded according to the laws applicable to all drivers, or to all drivers of a certain age, along with the dates when the corresponding laws became effective. Laws that applied only to drivers identified by factors other than age (e.g., based on crash involvement or traffic infractions) were not examined.

After compiling data from archival sources, questionnaires were developed for state driver licensing authorities to fill in any gaps and to confirm that all other information was correct by typing “OK” after each entry. The questionnaires were sent by e-mail to state Department of Motor Vehicles administrators, along with a cover letter explaining the research project. Completed questionnaires were obtained from representatives of all jurisdictions except Alabama, Connecticut, South Carolina, and the District of Columbia. Follow-up contacts were made by e-mail or telephone in cases where clarifications were required. Data from Oklahoma were excluded due to inability to ascertain the details of some laws. Data from the remaining 46 states were analyzed.

### 2.3 Statistical analysis

Associations between driver licensing policies and fatal crash involvement rates of older drivers were examined using population-averaged negative binomial regression. The dependent variable was the natural log of the number of drivers involved in fatal crashes in each quarter-year (January-March, April-June, July-September, October-December) in each state. The natural log of the age-specific state population (U.S. Census Bureau [2011]) was included as an offset variable. Age-specific estimates were obtained from interactions of licensing policy variables with indicator variables for each age group. Models were estimated using generalized estimating equations with a first-order autoregressive correlation structure (Hilbe [2011]). The robust variance estimator was used to correct variances for correlations of repeated measures from the same state.

All time-varying variables (*X*_*it*_) were decomposed into a cross sectional component $\left({\bar{X}}_{i}\right)$, which represented the mean value of *X*_*it*_ in state *i* over the entire study period, and a longitudinal component $\left(X_{\mathrm{it}}-{\bar{X}}_{i}\right)$, which represented the deviation at time *t* of each *X*_*it*_ from its mean value. Both components were included in the same model. The longitudinal component was used to estimate the effect of a policy change on rates of fatal crashes; the cross-sectional component was included to account for underlying differences between states that tended to have different policies during the study period, not to estimate the effects of policies.

Driver licensing policies examined were: the renewal period; mandatory in-person license renewal; mandatory vision, knowledge, and on-road driving testing; and mandatory vs. voluntary reporting laws for physicians. Licensing policies were coded according to those in effect on the first day of each quarter. Mandatory in-person renewal, vision tests, knowledge tests, and on-road driving tests were modeled as the proportion of all renewals at which each applied (0 = never, 1/3 = every third renewal, 1/2 = every other renewal, 1 = every renewal). Vision tests required in conjunction with in-person renewal were modeled as an effect modifier for in-person renewal; vision tests required outside of mandatory in-person renewal were modeled separately. Knowledge tests and on-road driving tests were modeled only as effect modifiers for in-person renewal, as neither was ever required when in-person renewal was not. Mandatory versus voluntary reporting laws for physicians were represented by a binary indicator variable.

The date when each individual crash-involved driver last renewed his or her license was not provided in the data, thus it was not always possible to ascertain what license renewal policies were in effect when a given crash-involved driver last renewed his or her license. Instead, the proportion of drivers in each age group who would have been subject to each respective licensing policy in a given year was estimated by modeling the renewal period as its reciprocal and dividing variables representing vision, knowledge, and on-road driving test requirements by the renewal period. For example, if the renewal period was 4 years and in-person renewal was required for every other renewal, then approximately 1/4 of drivers would have been required to renew their licenses each year $\left(\frac{1\;\mathrm{renewal}}{4\;\mathrm{years}}\right)$ and approximately 1/8 of drivers would have been required to renew their licenses in person each year $\left(\frac{1\;\mathrm{renewal}}{4\mathrm{years}}\times \frac{1\;\mathrm{in}‐\mathrm{person}\;\mathrm{renewal}}{2\;\mathrm{renewals}\;}\right)$. When the renewal period changed, the proportion of drivers who would have been required to renew their licenses each year would have been a function of both the previous renewal period and the current renewal period for several years following the change—this was modeled using weighted averages of the previous and current renewal periods. If any age-specific licensing policy took effect at an age other than the ages used to define age groups for analysis (e.g., at age 77), the corresponding variable was weighted by the proportion of the population of the age group to whom the requirement applied.

Potential confounders examined in preliminary models were per se blood alcohol concentration (BAC) laws, maximum Interstate highway speed limits, seatbelt use laws for drivers and front-seat passengers (Masten et al. [2011]; Insurance Institute for Highway Safety [2014]), state unemployment rates (Bureau of Labor Statistics [2013]), inflation-adjusted state per-capita personal income (Bureau of Economic Analysis [2013]), and inflation-adjusted state gasoline prices (Energy Information Administration [2012]). Models also included seasonal effects (dummy variables for quarters) and a linear time trend for each age group. Dummy variables for each year were included to account for nationwide yearly variation in rates of fatal crashes.

Because the effects of the driver licensing policies examined were often represented by functions of multiple policies (e.g., the effect of mandatory in-person renewal was modeled as the proportion of renewals that were required to be in-person divided by the renewal period), marginal standardization (Graubard and Korn [1999]; Cummings [2009]) was used to compute adjusted relative risks (aRR) for changes in each individual licensing policy given the distribution of the other licensing policies and confounders present in the population over the study period. For example, the effect of mandatory in-person renewal was estimated as follows. The fatal crash involvement rate in each state-quarter was predicted from the fitted model with the proportion of renewals required to be in-person set to 1, and again with it set to 0, each time using the actual observed values of all other explanatory variables in each state-quarter. The rates predicted with and without mandatory in-person renewal, respectively, were then averaged across all state-quarters. The average of the rates predicted with mandatory in-person renewal divided by the average of the rates predicted without mandatory in-person renewal is the aRR for mandatory in-person renewal. (Note that although the regression model took into account the phasing-in of each policy over time, the aRRs represent the effect of a policy that has been fully phased-in and is applicable to all drivers in a given age group, relative to the policy not being present at all.)

To account for other unmeasured factors that might have influenced rates of fatal crashes, the aRR for each licensing policy for each age group was divided by the aRR for the same licensing policy for drivers ages 40–54, who were used as a reference, yielding a ratio of relative risks (RRR).

Standard errors of aRRs and RRRs were estimated on the log scale using the delta method (Casella and Berger [2002]); the endpoints of the 95% confidence intervals of the logs of the aRR and RRR were exponentiated to obtain the 95% confidence intervals for the aRR and RRR.

To assess whether a potential confounder actually confounded the association of driver licensing policies with fatal crash involvement rates of older drivers, models were estimated with and without each potential confounder; the confounder was retained if the RRR for any licensing policy differed by 10% or more in the reduced model relative to its value in the full model, otherwise it was removed. Seatbelt laws; dummy variables for individual years; and interactions of age groups with BAC laws, unemployment rates, and per-capita personal income were thus removed.

The final model included age-specific seasonal effects and linear time trends; cross-sectional and longitudinal components of BAC laws, unemployment rates, and per-capita personal income; and interactions between each age group and longitudinal and cross-sectional components of gasoline prices, maximum Interstate highway speed limits, and all licensing policy variables. Analyses were performed using Stata version 13.1 (StataCorp LP, College Station, TX).

## 3 Results

### 3.1 License renewal period

During the study period, the driver’s license renewal periods in effect in states at various times ranged from 1 year to 12 years; Table 1 shows the distribution of state renewal periods by driver age for selected years. Many states had shorter renewal periods for younger drivers than for older drivers; the mean renewal period at the end of the study period was 5.7 years for drivers ages 40–54 and 4.4 years for drivers ages 85 years and older (Table 1). The average state renewal period increased over the course of the study period for all ages, though some states decreased their renewal periods for some or all drivers.

**Table 1:** **State driver licensing policies in effect on January 1 of selected years in relation to driver age, 46 U.S. states, 1986–2011**

	1986	1991	1996	2001	2006	2011
**Renewal period**	State renewal period, years Mean (minimum - maximum)					
Driver age (years)						
40-54	4.1 (3–6)	4.2 (3–6)	4.5 (3–12)	5.3 (4–12)	5.5 (4–12)	5.7 (4–12)
55-59	4.1 (3–6)	4.2 (3–6)	4.5 (3–12)	5.3 (4–12)	5.5 (4–12)	5.7 (4–12)
60-64	4.1 (3–6)	4.2 (3–6)	4.3 (3–6)	5.3 (4–12)	5.4 (4–12)	5.6 (4–12)
65-69	4.1 (2–6)	4.1 (2–6)	4.2 (2–6)	5.0 (4–8)	5.1 (4–8)	5.2 (4–8)
70-74	3.9 (2–6)	4.0 (2–6)	4.1 (2–6)	4.7 (2–8)	4.8 (2–8)	5.0 (2–8)
75-79	3.9 (2–6)	4.0 (2–6)	4.1 (2–6)	4.4 (1–8)	4.5 (1–8)	4.6 (1–8)
80-84	3.9 (2–6)	3.9 (2–6)	4.1 (2–6)	4.4 (1–8)	4.4 (1–8)	4.5 (1–8)
85+	3.9 (2–6)	3.9 (2–6)	4.1 (2–6)	4.4 (1–8)	4.4 (1–8)	4.4 (1–8)
**In-person renewal**	Number of states requiring renewal to be performed in-person: Every renewal (every other renewal, every third renewal)					
40-54	34 (6,1)	32 (8,1)	29 (10,2)	26 (13,2)	24 (17,2)	22 (20,1)
55-59	34 (6,1)	32 (8,1)	29 (10,2)	26 (13,2)	24 (17,2)	22 (20,1)
60-64	34 (6,1)	32 (8,1)	29 (10,2)	26 (13,2)	25 (16,2)	23 (19,1)
65-69	34 (6,1)	33 (7,1)	30 (9,2)	26 (13,2)	25 (16,2)	23 (19,1)
70-74	35 (5,1)	34 (7,0)	32 (8,1)	29 (11,1)	29 (13,1)	28 (15,0)
75-79	36 (5,1)	36 (6,0)	34 (7,1)	31 (10,1)	31 (11,1)	31 (12,0)
80-84	36 (5,1)	36 (6,0)	34 (7,1)	31 (10,1)	32 (10,1)	33 (10,0)
85+	36 (5,1)	36 (6,0)	34 (7,1)	31 (10,1)	32 (10,1)	33 (10,0)
Vision test*	Number of states requiring a vision test: At mandatory in-person renewal (outside of mandatory in-person renewal)					
40-54	34 (0)	35 (0)	35 (0)	35 (0)	35 (1)	36 (1)
55-59	34 (0)	35 (0)	35 (0)	35 (0)	36 (1)	37 (1)
60-64	34 (0)	35 (0)	35 (1)	35 (1)	36 (2)	37 (2)
65-69	34 (1)	35 (1)	35 (1)	35 (1)	37 (2)	38 (3)
70-74	34 (1)	35 (1)	35 (2)	35 (2)	37 (3)	38 (4)
75-79	34 (1)	35 (1)	35 (2)	35 (2)	37 (3)	38 (4)
80-84	34 (1)	35 (1)	35 (2)	35 (2)	37 (4)	38 (5)
85+	34 (1)	35 (1)	35 (2)	35 (2)	37 (4)	38 (5)
**Knowledge test**	Number of states requiring knowledge test at every in-person renewal					
40-54	2	2	2	1	0	0
55-59	2	2	2	1	0	0
60-64	2	2	2	1	0	0
65-69	2	2	2	1	0	0
70-74	2	3	3	2	1	1
75-79	3	4	4	3	1	1
80-84	3	4	4	3	1	1
85+	3	4	4	3	1	1
**On-road driving test**	Number of states requiring on-road driving test at every in-person renewal					
<70	0	0	0	0	0	0
70-74	1	0	0	0	0	0
75-79	3	3	3	3	2	2
80-84	3	3	3	3	2	2
85+	3	3	3	3	2	2
Reporting law for physicians*	Number of states in which reporting by physicians is mandatory					
	6	6	6	6	6	6

Alabama, Connecticut, District of Columbia, Oklahoma, and South Carolina were excluded due to missing data on history of laws.

*Data on vision testing & mandatory reporting law were excluded for Georgia for years 1986–1992.

After controlling for other licensing policies and potential confounders, a one-year reduction in the renewal period initially appeared to be associated with significant increases in the fatal crash involvement rates of drivers ages 60–64 and 70–74 years (Table 2, bottom panel). However, after accounting for concurrent changes in the fatal crash involvement rates of drivers in the reference group of drivers ages 40–54, changes in the renewal period were not associated with significant changes in fatal crash involvement rates of any of the older age groups, although RRRs approached statistical significance for the two oldest groups (Table 2, top panel).

**Table 2:** **Adjusted relative risks and ratios of relative risks, population-based fatal crash involvement rates of older drivers associated with changes in driver license renewal policies, United States, 1986–2011**

	Driver Age (years)							
	40-54	55-59	60-64	65-69	70-74	75-79	80-84	85+
	**Ratio of relative risks (95% confidence interval)**							
**Renewal period (1 year reduction)**	Reference	0.99 (0.92–1.08)	1.03 (0.97–1.10)	1.00 (0.93–1.08)	1.05 (0.95–1.17)	0.96 (0.88–1.06)	0.94 (0.88–1.00)	0.95 (0.89–1.02)
**In-person renewal**	-	1.01 (0.77–1.33)	0.86 (0.66–1.11)	0.85 (0.65–1.10)	0.92 (0.69–1.23)	0.74 (0.48–1.15)	0.91 (0.67–1.24)	0.69 (0.48–0.97)
**Vision testing (overall)**	-	1.01 (0.86–1.20)	1.08 (0.89–1.29)	1.06 (0.92–1.23)	1.00 (0.84–1.19)	1.05 (0.79–1.40)	0.93 (0.80–1.07)	0.93 (0.78–1.10)
**Vision testing (at in-person renewal)**	-	1.01 (0.83–1.23)	1.13 (0.93–1.38)	1.16 (0.96–1.39)	0.98 (0.81–1.18)	1.15 (0.85–1.56)	0.98 (0.80–1.19)	1.06 (0.84–1.32)
**Vision testing (without in-person renewal)**	-	1.03 (0.76–1.39)	0.98 (0.67–1.44)	0.91 (0.70–1.19)	1.06 (0.70–1.61)	0.85 (0.41–1.76)	0.81 (0.63–1.03)	0.64 (0.49–0.85)
**Knowledge test (at in-person renewal)**	-	0.83 (0.55–1.26)	0.98 (0.74–1.28)	0.98 (0.64–1.51)	0.95 (0.76–1.19)	0.89 (0.70–1.13)	0.93 (0.74–1.17)	0.97 (0.76–1.24)
**On-road driving test (at in-person renewal)** ^**a**^	-	-	-	-	-	-	-	-
**Mandatory reporting law for physicians** ^**b**^	-	1.03 (0.87–1.23)	1.01 (0.86–1.19)	0.98 (0.83–1.16)	1.03 (0.88–1.21)	1.00 (0.86–1.16)	0.98 (0.84–1.15)	0.96 (0.82–1.14)
	**Adjusted relative risk (95% confidence interval)**							
**Renewal period (1 year reduction)**	1.03 (0.98–1.09)	1.03 (0.97–1.09)	1.07 (1.02–1.11)	1.03 (0.98–1.09)	1.09 (1.00–1.19)	1.00 (0.92–1.08)	0.97 (0.93–1.01)	0.99 (0.95–1.03)
**In-person renewal**	1.05 (0.84–1.31)	1.06 (0.91–1.25)	0.90 (0.79–1.03)	0.89 (0.78–1.02)	0.97 (0.80–1.16)	0.78 (0.54–1.14)	0.96 (0.77–1.19)	0.72 (0.55–0.94)
**Vision testing (overall)**	0.96 (0.86–1.07)	0.97 (0.85–1.10)	1.03 (0.89–1.20)	1.02 (0.93–1.12)	0.96 (0.83–1.10)	1.01 (0.78–1.31)	0.89 (0.80–0.98)	0.89 (0.77–1.02)
**Vision testing (at in-person renewal)**	0.94 (0.80–1.11)	0.95 (0.85–1.06)	1.06 (0.95–1.19)	1.09 (1.00–1.18)	0.92 (0.84–1.01)	1.08 (0.83–1.40)	0.92 (0.82–1.03)	0.99 (0.85–1.16)
**Vision testing (without in-person renewal)**	0.99 (0.87–1.12)	1.01 (0.77–1.34)	0.97 (0.68–1.39)	0.90 (0.71–1.14)	1.05 (0.70–1.56)	0.84 (0.41–1.72)	0.80 (0.65–0.98)	0.64 (0.50–0.81)
**Knowledge test (at in-person renewal)**	1.13 (0.93–1.38)	0.94 (0.65–1.36)	1.11 (0.92–1.33)	1.11 (0.76–1.62)	1.07 (0.97–1.18)	1.01 (0.89–1.15)	1.05 (0.94–1.18)	1.10 (0.94–1.27)
**On-road driving test (at in-person renewal)**	-	-	-	-	1.00 (0.93–1.08)	0.96 (0.85–1.07)	1.09 (0.97–1.23)	1.02 (0.92–1.14)
**Mandatory reporting law for physicians** ^**b**^	1.07 (0.94–1.21)	1.10 (0.99–1.24)	1.08 (0.98–1.20)	1.05 (0.94–1.17)	1.11 (1.01–1.21)	1.07 (0.98–1.16)	1.05 (0.96–1.16)	1.03 (0.93–1.14)

Data: Fatal crashes: National Highway Traffic Safety Administration ([2013]); population: (U.S. Census Bureau [2011]); driver licensing policies: surveys of states.

Adjusted relative risks estimated using population-averaged negative binomial regression, adjusted for: age-specific seasonal effects and linear time trends; per se blood alcohol concentration laws; unemployment rates; per-capita personal income; age-specific effects of gasoline prices, maximum Interstate highway speed limits, all other variables in table, and within-state means of all time-varying variables; standardized to the observed distribution of all variables except row variable.

Alabama, Connecticut, District of Columbia, Oklahoma, and South Carolina were excluded for all years; Georgia was excluded for years 1986–1992.

a. Ratios of relative risks could not be computed for on-road driving tests because no state required drivers in the reference group to pass an on-road driving test at routine in-person license renewal at any time during the study period.

b. Adjusted relative risk for mandatory reporting law for physicians represents ratio of adjusted fatal crash involvement rate with versus without mandatory reporting law as no state enacted or repealed a mandatory reporting law during the study period; all other adjusted relative risks represent changes in adjusted fatal crash involvement rates associated with changes in corresponding policies.

### 3.2 In-person renewal

Although most states required either every license renewal or every other renewal to be conducted in person, older drivers were more likely than younger drivers to be required to appear in person for every renewal. This was especially true toward the end of the study period, as several states began to allow younger drivers to renew their licenses by mail or online but continued to require the oldest drivers to renew in person (Table 1).

Implementing a requirement for drivers to renew their licenses in person was associated with a 28% reduction in fatal crash involvement rates of drivers ages 85 years and older (aRR: 0.72, 95% CI: 0.55–0.94) after controlling for potential confounders as well as other licensing requirements sometimes present in conjunction with in-person renewal (Table 2, bottom panel). After accounting for concurrent changes in the fatal crash involvement rates of drivers ages 40–54, the estimated reduction in fatal crash involvement rates of drivers ages 85 years and older was 31% (RRR: 0.69, 95% CI: 0.48–0.97). Estimated effects were smaller (RRRs were closer to 1) and were not statistically significant for younger age groups.

### 3.3 Vision test

Most states required all drivers to pass a vision test at every in-person license renewal irrespective of age, though a small number of states required only drivers older than a certain age to pass a vision test, and as noted previously, many states required older drivers to renew their license more frequently in general and to do so in-person at a higher proportion of renewals (Table 1). While most required vision testing was required and performed in conjunction with mandatory in-person license renewal, 6 states required drivers to pass a vision test even when renewal was not required to be in-person (e.g., allowed drivers to renew their license by mail or online but in such cases required the driver to submit a vision report from a healthcare provider).

Overall, changes in requirements for drivers to pass a vision test when renewing their license were not associated with statistically significant changes in rates of fatal crash involvement for older drivers. However, when in-person renewal was not required, implementing a requirement for drivers to pass a vision test was associated with a significant reduction in fatal crash involvement rates for drivers ages 85 years and older (RRR: 0.64, 95% CI: 0.49–0.85) (Table 2). When in-person renewal was required, and vision testing was required as a component of in-person renewal, the vision test was not associated with any additional reduction in rates of fatal crashes beyond that associated with in-person renewal alone.

### 3.4 Knowledge test

Only four states required any drivers to pass a knowledge test (beyond simple recognition of road signs) at routine in-person license renewal at any point during the study period. Hawaii and Michigan applied the requirement to all drivers, California to drivers ages 70 and older, and Indiana to drivers ages 75 and older. Hawaii, Indiana, and Michigan had their requirements in effect at the beginning of the study period and repealed them in 1997, 2003, and 2004, respectively, providing some data with which to assess the effect of the policy change. California’s knowledge testing requirement took effect in the second year of the study period and was retained throughout, providing very little data with which to examine the effect of the policy change.

There was no evidence that requiring a driver to pass a knowledge test at routine in-person license renewal was associated with any reduction in fatal crash involvement rates beyond that associated with mandatory in-person renewal alone. The aRR for requiring a knowledge test was larger than 1 for all age groups except ages 55–59; however, none even approached statistical significance, nor did the RRRs (Table 2).

### 3.5 On-road driving test

Only three states—Illinois, Indiana, and New Hampshire required drivers above a specified age to pass an on-road driving test at routine license renewal at any point during the study period. Illinois had a road test requirement that applied to drivers ages 69 years and older at the beginning of the study period, changed the requirement to take effect at age 75 in 1989, and it remained in effect for the remainder of the study period. New Hampshire had a requirement that applied to drivers ages 75 and older for all but the final five months of the study period. Indiana required drivers ages 75 and older to pass an on-road driving test at routine license renewal from the beginning of the study period through 2004, and then repealed the requirement in 2005.

Implementing a requirement for drivers to pass an on-road driving test at routine in-person license renewal was not associated with significant changes in fatal crash involvement rates for drivers in any of the age groups examined. RRRs could not be computed because drivers in the reference group aged 40–54 were never required to pass an on-road driving test at routine in-person license renewal in any state; however, aRRs provided no significant evidence of any effect (Table 2).

### 3.6 Mandatory reporting laws

Although six states required physicians to report patients to the licensing authority under some conditions during the study period, no state enacted or repealed such a law, thus it was not possible to assess the effect of enacting or repealing such a law in the same manner as was done for other laws and policies examined in this study. However, in adjusted cross-sectional comparisons, fatal crash involvement rates of older drivers were not significantly higher or lower in states with mandatory reporting laws for physicians compared with states in which reporting was voluntary.

## 4 Discussion

This study investigated the relationship between several specific state driver license renewal policies and the fatal crash involvement rates of older drivers. Results showed that requiring drivers to renew their licenses in person was associated with significant reductions in population-based fatal crash involvement rates for drivers ages 85 years old and older, a result also reported by Grabowski et al. ([2004]). Results also showed that when in-person renewal was not required, requiring drivers to pass a vision test was associated with similar reductions in rates of fatal crashes among drivers ages 85+. However, when in-person renewal was required, requiring a vision test at in-person renewal was not associated with any further reduction in rates of fatal crashes. The length of the renewal period, requirements to pass a knowledge test or an on-road driving test, and mandatory reporting laws for physicians were not found to be significantly associated with rates of fatal crashes.

Previous literature on the relationship between vision testing and rates of fatal crashes has been mixed. Levy *et al.* ([1995]) and Shipp ([1998]) both analyzed data from all US states from the late 1980’s and found that older drivers had significantly lower population-based fatal crash involvement rates in states that required vision testing at routine license renewal than in states that did not. However, the methods used by Shipp only investigated cross-sectional differences in fatal crash involvement rates between states with different laws. The methods of Levy et al. made use of both cross-sectional and longitudinal variation; however, there was very little longitudinal change in state vision testing policies over the period of Levy’s study, thus those results would have been weighted heavily toward cross-sectional differences in crash rates of states with different vision testing policies than longitudinal changes in crash rates associated with changes in vision testing policies. Grabowski et al. ([2004]) used methods that removed cross-sectional variation in policies from the model and only examined longitudinal changes in policies, and like the current study, found no significant overall association between enacting or repealing of vision testing requirements and rates of fatal crashes of older drivers. The current study used cross-sectional variation in policies not to estimate their effects but rather to control for underlying differences between states, and used longitudinal changes in policies to estimate their effects. Like Grabowski et al., the current study found no association between changes in vision testing policies overall and rates of fatal crashes.

Unlike previous studies, however, the current study distinguished between vision testing required as a component of mandatory in-person renewal versus vision testing required in the absence of mandatory in-person license renewal. The finding that when in-person renewal was not required, mandatory vision testing was associated with a reduction in fatal crash involvement rates similar to that associated with in-person renewal for drivers ages 85 and older is a new finding not reported in previous studies.

McGwin et al. ([2008a]) investigated the impact of a Florida law requiring drivers ages 80 years and older to submit a vision report from a healthcare provider if renewing their license by mail or online, and found that fatal crashes of drivers ages 80 and older decreased by 17% after the law took effect in 2004, whereas no such change in fatal crashes was observed in two neighboring states that did not change their vision testing policies. (At the time of the study, every third renewal was required to be in-person and included a vision test; other renewals could be performed by mail or online and previously did not require a vision test.) These results were generally consistent with the finding of the current study that requiring a vision test when in-person renewal was not required was associated with reductions in rates of fatal crashes of drivers ages 85 years and older.

The finding that simply requiring drivers ages 85 years or older to renew their license in person, *or* to pass a vision test if not renewing in person, was associated with a significant reduction in their fatal crash involvement rates, but requiring drivers to pass a vision test as a component of mandatory in-person renewal was not associated with further reductions, was unexpected and potentially important. One speculative but plausible hypothesis is that requiring older drivers to interact with a professional when renewing their license—whether a licensing professional or a healthcare professional—may provide as much benefit as requiring them to undergo a specific test or tests. It is possible that license office staff and/or healthcare providers may be able to identify potentially-unsafe older drivers quickly during these encounters and refer those specific drivers for further evaluation, as opposed to mandating that all drivers over a certain age take vision tests, knowledge tests, or on-road driving tests. Meuser et al. ([2009]) studied drivers referred for medical review in the state of Missouri and found that 27% of them were referred by license office staff; the most common reasons for referral were concerns about ability to walk, apparent confusion, and general frailty (i.e., not vision problems). It is also possible, however, that the seemingly-simple requirement to visit the licensing office or pass a vision test administered by a healthcare provider might deter a substantial proportion of drivers from attempting to renew their license in the first place. McGwin et al. ([2008b]) found that after Florida enacted a requirement for drivers ages 80 years and older to pass a vision test if renewing their license by mail or online, 93% of those who attempted to renew their license succeeded, but 20% of those eligible for renewal chose not to do so. More research is needed to understand the mechanisms by which the reductions in fatal crashes associated with mandatory in-person renewal and vision testing outside of mandatory in-person renewal come about.

One might expect that shortening the renewal period, thus requiring drivers to interact with the licensing agency more frequently, would reduce the prevalence of unsafe drivers on the road and decrease overall crash rates. However, this study found no statistically significant association between changes in the renewal period and changes in adjusted fatal crash involvements for any age group. It did appear, however, that shorter renewal periods might have been associated with reduced rates of fatal crashes for drivers ages 80–84 and 85+, as although results did not attain statistical significance, RRRs for a one-year reduction in the renewal period were 0.94 and 0.95, respectively, for these two groups, suggestive of possible benefits that the study lacked the statistical power to detect. A *post hoc* Wald *χ*^2^ test of homogeneity of the RRRs suggested that the effect of the renewal period varied significantly by age (P = 0.025), and a *post hoc* calculation of the effect of a one-year reduction in the renewal period for drivers ages 80 years and older (i.e., 80–84 and 85+ combined) yielded a nearly-significant RRR of 0.95 (95% CI: 0.89–1.00, P = 0.06).

### 4.1 Limitations

This study had low statistical power to detect effects of some policies. Although the study examined data from 46 states spanning 26 years, some of the policies that the study sought to examine were subject to very little change over time. While most states changed their renewal period, in-person renewal requirements, and vision testing requirements for some or all drivers at some point during the study period, there were few changes in requirements for drivers to pass a knowledge test or on-road driving test, which substantially limited the study’s ability to detect any possible impact of these policies. No state implemented or repealed a mandatory reporting law for physicians during the study period, thus the effect of mandatory reporting laws was based on comparison of adjusted rates of fatal crashes in states with versus without laws—a cross-sectional comparison—which is more susceptible to bias than the longitudinal comparisons on which estimates of the effects of other policies were based. Thus, although this study produced no evidence of safety benefits associated with requiring drivers to pass a knowledge test or an on-road driving test, or of requiring physicians to report patients to the licensing authority, this study should not be interpreted as strong evidence against the possibility that such policies could be beneficial.

It was not always possible to determine what set of license renewal policies would have been in effect at the time when a particular crash-involved driver last renewed his or her license, because the date at which a given driver last renewed his or her license is not reported in available data on fatal crashes. Instead, the proportion of drivers in a state who would have been affected by a particular law or policy in a given year was estimated from the age distribution of the state’s population and the length of the license renewal period. While this estimation procedure is likely to introduce some error, the error is expected to be relatively small and mostly random. However, if the estimated proportion of drivers affected by a policy were consistently biased in a particular direction, this could lead to over- or under-estimation of the impact of the corresponding license renewal policies.

The type of modeling used in this study may fail to capture the unique impact of a policy implemented in a particular manner in one state and implemented differently in another. Assessing the relative strength, rigor, or difficulty of various policies as implemented in various states (e.g., a more lenient versus more stringent cutoff score for passing a mandatory vision test) was beyond the scope of this study. In-depth studies of the impacts of policy changes in individual states would be required to gain further insight into how the specific details of a particular state’s implementation of a law or policy influence its impact.

Modeling the effects of laws and policies implemented in different states, at different times, sometimes in different forms, is very complex. Many other factors besides driver licensing policies influence rates of fatal crashes and could bias the results of such a study. Substantial effort was made to control for other variables that might confound the relationship between driver license renewal policies and rates of fatal crashes. In addition, changes in rates of fatal crashes among older drivers were further adjusted by computing ratios relative to corresponding changes in rates of fatal crashes among a reference group of drivers ages 40–54, whose crash rates were not expected to have been affected to any meaningful degree by the types of policies examined here, to attempt to control for any other factors that might also have influenced fatal crash involvement rates of older drivers. Notably, most results were similar with this adjustment (Table 2, top panel, RRRs) or without it (Table 2, bottom panel, aRRs). However, bias could still be present if the effects of omitted variables varied by age.

Finally, this study could not determine the mechanism by which a particular law or policy affected the fatal crash involvement rates of older drivers. As discussed previously, the observed results could be attributable to the effective identification of potentially-unsafe drivers at in-person renewal, or alternatively, they could be due to premature driving cessation by some drivers who were still able to drive quite safely. Unfortunately, this question could not be addressed due to data limitations. Specifically, valid historical data on age-specific driver licensing rates by state are not available for most states. For example, in the state-by-state driver licensing data published by the Federal Highway Administration (FHWA), many states in past years did not report detailed distributions of the number of licensed older drivers by age, in which case the FHWA would estimate them based on data from previous years (Federal Highway Administration [2006]), thus rendering investigation of the relationship between changes in driver licensing policies and the licensing rates of specific subgroups of older drivers (or rates of fatal crashes per licensed driver) unreliable. In-depth studies of policy changes in individual states could contribute substantially to knowledge of the mechanisms by which policies impact older drivers by examining mobility outcomes (e.g., the proportion of drivers who attempt to renew their license as opposed to allowing it to expire, the proportion of those attempting to renew who are successful, and the reasons why those who do not successfully renew their license do not do so), in addition to safety outcomes such as crash involvement. The studies by McGwin et al. of the safety impacts (McGwin et al. [2008a]) and mobility impacts (McGwin et al. [2008b]) of Florida’s vision testing requirement for drivers ages 80 years and older are examples of state-specific studies that provide such needed in-depth analysis.

## 5 Conclusions

Mandatory in-person license renewal was associated with significant reductions in population-based fatal crash involvement rates of drivers ages 85 years and older, confirming the results of previous studies. When in-person renewal was not required, requiring drivers to pass a vision test in order to renew their license was associated with similar reductions; however, requiring a vision test at in-person renewal when in-person renewal was already required was not found to yield any additional reduction in rates of fatal crashes beyond that associated with in-person renewal alone. There was weak suggestive evidence that requiring drivers to renew their licenses more frequently might yield small reductions in the fatal crash involvement rates of the oldest drivers; however, this was not statistically significant. There was no evidence that requiring drivers to pass a knowledge test or an on-road driving test, or requiring physicians to report drivers to the licensing authority under specific circumstances as opposed to allowing them to do so voluntarily, reduced rates of fatal crashes; however, this study had very limited ability to detect any such benefits had they existed, and thus should not be taken as strong evidence against the possibility that such policies could improve safety.

More research is needed to understand the mechanisms by which requiring drivers to renew their licenses in person or pass a vision test outside of in-person renewal influences population-based fatal crash involvement rates. This study could not determine whether these effects were primarily accomplished through removing unsafe drivers from the driving population or whether these policies fostered some degree of premature driving cessation among drivers who were still able to drive safely. Given the absence of valid historical data on driver licensing rates in relation to driver age over a long period of time from a large number of states, in-depth studies of the effects of policy changes in individual states will be needed to elucidate answers to these questions.
